# Biomechanical stress regulates mammalian tooth replacement

**DOI:** 10.15698/cst2020.03.215

**Published:** 2020-02-18

**Authors:** Xiaoshan Wu, Songlin Wang

**Affiliations:** 1 Beijing Key Laboratory of Tooth Regeneration and Function Reconstruction, Capital Medical University School of Stomatology, Beijing 100050, China.; 2 Department of Oral and Maxillofacial Surgery, Xiangya Hospital, Central South University, Changsha 410008, China.; 3 Department of Biochemistry and Molecular Biology, Capital Medical University School of Basic Medical Sciences, Beijing 100069, China.

**Keywords:** biomechanics, organ replacement, stress, Wnt signaling

## Abstract

Cyclical renewal of integumentary organs, including hair, feathers, and teeth occurs throughout an organism's lifetime. Transition from the resting to the initiation stage is critical for each cycle, but the mechanism remains largely unknown. Humans have two sets of dentitions—deciduous and permanent—and tooth replacement occurs only once. Prior to eruption of the permanent tooth (PT), the successional dental lamina (SDL) of the PT can be detected as early as the embryonic stage, even though it then takes about 6–12 years for the SDL to develop to late bell stage. Little is known about the mechanism by which resting SDL transitions into the initiation stage inside the mandible. As a large mammal, the miniature pig, which is also a diphyodont, was a suitable model for our recent study (EMBO J (2020)39: e102374). Using this model, we found that the SDL of PT did not begin the transition into the bud stage until the deciduous tooth (DT) began to erupt.

The DT eruption released a biomechanical stress of about 3-20 kPa inside the mandible. By culturing the mandible slice *in vitro*, the SDL of the PT immediately transitioned into the bud stage. However, the initiation was inhibited when an external force more than 3 kPa was applied to compensate for the stress loss *in vitro*. No change was observed in the expression level of the integrin β1-ERK1-RUNX2 axis in the mesenchyme between the DT and the PT in response to mechanical stress. Similar molecular expression patterns were observed in human tooth germs. Overexpression of RUNX2 lentiviral-mediated transfection inhibited the PT development. RUNX2 acts upstream of wingless/integrated (Wnt) signaling in the mesenchyme between DT and PT. The release of the biomechanical stress induced downregulation of RUNX2-Wnt signaling in the mesenchyme and upregulation of Wnt signaling in the epithelium of PT, thereby triggering PT initiation. Our study identified biomechanical stress-associated Wnt modulation as an initiator of organ renewal, and this finding could be valuable for future study of integumentary organ regeneration.

Little is known about the morphology of the SDL of the PT of large mammals in the early stages. Our study demonstrated that the SDL of the permanent canine (PC) was first connected to the outer enamel epithelium of the deciduous canine (DC) and then separated. The epithelial stem cell marker, Sox2, was expressed at the tip of the SDL. After the separation at E60, the space between DC and SDL of PC was occupied by the mesenchyme of the dental follicle (**Fig. 1**). Importantly, we found that the SDL remained in the resting stage until the beginning of the bud stage, when the DC started erupting at E90. This phenomenon was found to be similar in all tooth positions in miniature pig.

**Figure 1:**
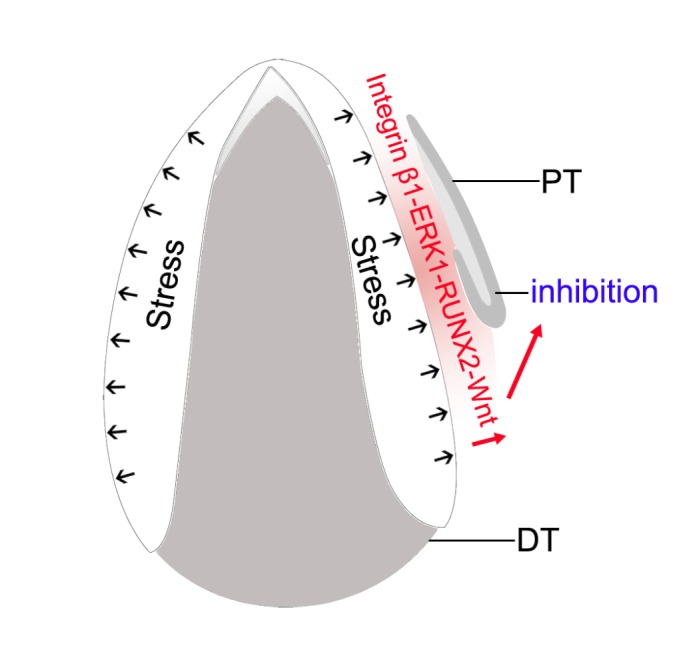
FIGURE 1: Biomechanical stress associated Integrin β1-ERK1-RUNX2-Wnt pathway inhibits the development of SDL of permanent tooth before the eruption of deciduous tooth. PT: permanent tooth; DT: deciduous tooth.

The differential growth rate, which was observed between the DC and the alveolar socket, may result in the generation of mechanical stress inside the mandible. Using micro-surgery and finite element analysis software (ANSYS), we established a “cup” model to estimate the magnitude of stress inside the mandible and found that stress ranges from 3 to20 kPa in the E60 mandible. To investigate the effect of biomechanical stress on PC initiation, we first dissected the mandible slice containing both DC and PC, to release the biomechanical stress inside the mandible. After *in vitro* culture, the SDL of PC transitioned into the cap stage in two days. However, the development of the SDL was inhibited when extra force was applied using Flexcell FX-5000 Compression System to compensate for the loss of stress.

With or without biomechanical stress, the integrin β1-ERK1-RUNX2 pathway was expressed strongly or weakly in the mesenchyme between DC and SDL of PC, respectively. This finding was confirmed both *in viv*o and *in vitro* experiments (**Fig. 1**). We found that *RUNX2* overexpression inhibited the PC initiation. In contrast, *RUNX2* knockdown led to the development of SDL.

Similar to the SDL of reptiles, the activation of canonical Wnt signaling was observed in the tip of the SDL when the SDL grew into the bud stage. However, the canonical Wnt signaling was only observed in the mesenchyme between the DC and SDL of PC prior to initiation. Thus, the phenomenon of “Wnt translocation” was observed between the mesenchyme and the epithelium during the initiation process. Interestingly, the expression pattern of Wnt signaling in the mesenchyme was similar to that of RUNX2 before eruption. We then proved that biomechanical stress also modulated Wnt signaling in the mesenchyme, and RUNX2 acted upstream of Wnt signaling (**Fig. 1**).

To improve our understanding of PT initiation, a number of questions will have to be addressed: i) Does the Wnt signaling in the mesenchyme repress the initiation of SDL of PT? ii) What is the mechanism of “Wnt translocation” between mesenchyme and epithelium? iii) What is the role of the Wnt ligand in this process? The mechanism of biomechanical stress-associated Wnt modulation between organ epithelium and the surrounding mesenchyme is expected to be valuable for future studies on integumentary organ regeneration.

## Abbreviations:

DC: – deciduous canine;
DT: – deciduous tooth;
PT: – permanent tooth;
SDL: – successional dental lamina.

